# Comprehensive analyses of genomic features and mutational signatures in adenosquamous carcinoma of the lung

**DOI:** 10.3389/fonc.2022.945843

**Published:** 2022-09-14

**Authors:** Hongbiao Wang, Jun Liu, Sujuan Zhu, Kun Miao, Zhifeng Li, Xiaofang Qi, Lujia Huang, Lijie Guo, Yan Wang, Yuyin Cai, Yingcheng Lin

**Affiliations:** ^1^ Medical Oncology Session No.1, Cancer Hospital of Shantou University Medical College, Shantou, China; ^2^ Department of Thoracic Surgery, The First People’s Hospital of Yunnan Province, Kunming, China; ^3^ Department of Medical Oncology, The First People’s Hospital of Yunnan Province, Kunming, China; ^4^ Medical Department, OrigiMed Co., Ltd, Shanghai, China; ^5^ Department of Thoracic Surgery, The Second Affiliated Hospital of Kunming Medical University, Kunming, China

**Keywords:** adenosquamous carcinoma of the lung, next-generation sequencing, mutational signature, TMB, EGFR mutation

## Abstract

Adenosquamous carcinoma (ASC) of the lung is a relatively rare tumor with strong aggressiveness and poor prognosis. The analysis of mutational signatures is becoming routine in cancer genomics and has implications for pathogenesis, classification, and prognosis. However, the distribution of mutational signatures in ASC patients has not been evaluated. In this study, we sought to reveal the landscape of genomic mutations and mutational signatures in ASC. Next-generation sequencing (NGS) technology was used to retrieve genomic information for 124 ASC patients. *TP53* and *EGFR* were the most prevalent somatic mutations observed, and were present in 66.9% and 54.8% of patients, respectively. *CDKN2A* (21%), *TERT* (21%), and *LRP1B* (18.5%) mutations were also observed. An analysis of gene fusion/rearrangement characteristics revealed a total of 64 gene fusions. The highest frequency of variants was determined for *ALK* fusions, with six *ALK-EML4* classical and two intergenic *ALK* fusions, followed by three *CD74-ROS1* fusions and one *ROS1-SYN3* fusion. *EGFR* 19del (45.6%), and *EGFR* L858R (38.2%) and its amplification (29.4%) were the top three *EGFR* mutations. We extracted mutational signatures from NGS data and then performed a statistical analysis in order to search for genomic and clinical features that could be linked to mutation signatures. Amongst signatures cataloged at COSMIC, the most prevalent, high-frequency base changes were for C > T; and the five most frequent signatures, from highest to lowest, were 2, 3, 1, 30, and 13. Signatures 1 and 6 were determined to be associated with age and tumor stage, respectively, and Signatures 22 and 30 were significantly related to smoking. We additionally evaluated the correlation between tumor mutational burden (TMB) and genomic variations. We found that mutations *ARID2*, *BRCA1*, and *KEAP1* were associated with high TMB. The homologous recombination repair (HRR) pathway-related gene mutation displayed a slightly higher TMB than those without mutations. Our study is the first to report comprehensive genomic features and mutational signatures in Chinese ASC patients. Results obtained from our study will help the scientific community better understand signature-related mutational processes in ASC.

## Introduction

Adenosquamous carcinoma (ASC) of the lung, a relatively rare subtype of non-small cell lung cancer (NSCLC), is defined as a malignancy containing components of lung adenocarcinoma (ADC) and lung squamous cell carcinoma (SCC) (1). ASC is a cancer containing both SCC and ADC components, each of which accounts for at least 10% of tumors, as outlined in the currently valid 2015, 5^th^ edition of the World Health Organization (WHO) classification system (2). Reports indicate that ASC yields a worse prognosis than other types of NSCLC resistant to treatments with adjuvant chemotherapy; and ASC patients are more likely to develop local recurrence or distant metastasis as compared to patients having other histological types of NSCLC (3, 4). To date, no uniform standard chemotherapy regimen for ASC exists, and treatment regimens currently rely on NSCLC guidelines (5). At present, surgical resection is the only effective means for curing ASC. Therefore, understanding ASC’s molecular characteristics, as well as potential drugs and immunotherapies, is an urgent need.

Accumulating molecular evidence suggests that ASC may be monoclonal in origin. Similar driver oncogenes such as *EGFR* and *KRAS* mutations have been observed in both adenoid cystic carcinoma (ACC) and squamous cell carcinoma components (SCCC). *EGFR*-tyrosine kinase inhibitor (TKI) therapy has been demonstrated to be effective in selected patients with advanced pulmonary ASC (6, 7). However, continued research is needed to identify additional cancer-related genetic mutations, as well as *EGFR* mutations, and corresponding targeted agents or combination therapies that will improve pulmonary ASC outcomes. Given current limitations and their implications for clinical management, clearly, a more comprehensive genomic study of ASC is required.

ASC is a striking example of a morphologically dichotomous tumor whose genomic landscape has yet to be systematically probed. An in-depth study of the molecular characteristics of ASC will not only help the scientific community to better understand disease features, including the phenotype switching of lung cancer, the origin of tumor development, and tumor heterogeneity, but will also contribute to the development of individualized treatments. To date, research on ASC has generally involved small sample sizes and a focus on mutation frequencies and clinical features. To address gaps in knowledge, we evaluated genomic variations, mutational signature distributions, and the tumor mutational burden (TMB) index in patients diagnosed with lung ASC. We also compared genetic signatures to clinicopathologic features.

## Materials and methods

### Patients and samples

A total of 124 ASC patients who underwent surgical resection from the Cancer Hospital of Shantou University Medical College, The Second Affiliated Hospital of Kunming Medical University and First People’s Hospital of Yunnan Province were enrolled in our study. For the purpose of validation, pathological diagnoses of lung ASC were re-reviewed, according to the 5^th^ edition of WHO for tumor classification, by two experienced pathologists. Based on WHO criteria, each component of ADC and SCC represented at least 10% of tumor cells. Complete medical records included patient age, gender, smoking history, immunohistochemistry results, pathological reports, operation time and surgical approach, and medication records. All patents signed informed consent forms. Both tumor tissue and matched normal blood samples were collected from each patient. All procedures performed in our study involving human participants were accomplished in accordance with the Declaration of Helsinki (as revised in 2013). Our study was approved by the Committee for Ethical Review of Research at Cancer Hospital of Shantou University Medical College (2021090).

### Next-generation sequencing

Formalin-fixed, paraffin-embedded (FFPE) tumor tissues and matched blood samples were obtained from three hospitals mentioned above. At least 50 ng of cancer tissue DNA was extracted from the 40 mm FFPE and from blood samples using a DNA Extraction Kit (QIAamp DNA FFPE Tissue Kit, Qiagen, Venlo, Netherlands) for subsequent targeted NGS-based genomic testing (OrigiMed, Shanghai, China). All of the coding exons for cancer-related genes and selected introns for parts of targeted genes frequently rearranged in solid tumors were captured, using the custom hybridization capture panel, and then sequenced using the Illumina NextSeq-500 Platform (Illumina, CA, USA). For FFPE samples, the sequencing depth mean coverage was 900x (minimum 700x), while for matched blood samples the sequencing depth was 300x. Genomic profiles were analyzed using 450 gene-targeted NGS.

### The mutational signature analysis

Mutation signatures were classified, and the proportion of each mutation signature was calculated using the deconsrtuctSigs package (8). Input data consisted of a data frame containing mutational data for the tumor sample set which included the genomic position, the base change for each mutation, and the sample identifier. Mutational signature classification was obtained using COSMIC Mutational Signature, in the same manner as described in past studies (9, 10).

### The TMB calculation and PD-L1 analysis

TMB was calculated by counting somatic mutations, including coding base substitutions and indel mutations per megabase (muts/Mb) of genome examined, and by excluding known hotspot mutations in oncogenic drivers and known germline alterations within the single nucleotide polymorphism database (dbSNP) (11). Using previous studies as a guide, the threshold for high TMB was set as ten.

Immunohistochemical staining for FFPE tissue sections was performed using anti-PD-L1 antibodies (clone 22C3, Cat # M3653, DAKO, Agilent, CA, USA). Dilutions (28-8 1:300; 22C3 1:50) of primary antibodies were used for antigen detection. All slides were counterstained with hematoxylin. The PD-L1 tumor proportion score (TPS), which is the percentage of tumor cells showing partial or complete membrane staining, was determined; and samples were classified as negative, low-positive, or high-positive (TPS of <1%, 1%–49%, and ≥50%, respectively) (12).

### Statistical analyses

Statistical analyses in this study were performed using R Foundation for Statistics Computing, R script (v3.6.0). A chi-square test and a Fisher’s exact test were performed when a rate or percentage were compared for significance. Comparisons were determined between mutational signatures that were adjusted for age, sex, stage, and smoking. TMB comparisons between different groups were investigated by employing a Wilcoxon rank-sum test or a *t* test. For all analyses, a *p*-value less than 0.05 was considered statistically significant.

## Results

### Patient characteristics and molecular profiling

Patient characteristics, including age at diagnosis, gender, smoking status, stage, and family history, are provided in **Table 1**. Amongst the 124 patients investigated, 77 (62.10%) were male and 47 (37.90%) were female. Seventy-four (74, 59.68%) were < 65 years old and 50 (40.32%) patients were ≥ 65 years old. Forty-three (34.68%) patients smoked, and 52 (41.94%) patients never smoked. Thirty (30, 24.19%) patients had a family history of cancer. The remaining 94 (75.81%) patients had no family history of cancer.

**Table 1:** Patients’ clinicopathologic characteristics.

Variable	Classification	Results, n (%)
Age		
	< 65	74 (59.68%)
	≥ 65	50 (40.32%)
Gender		
	Male	77 (62.10%)
	Female	47 (37.90%)
Smoking status		
	Smokers	43 (34.68%)
	Non-smokers	52 (41.94%)
	Unknown	29 (23.39%)
Stage		
	I	15 (12.10%)
	II	17 (13.71%)
	III	30 (24.19%)
	IV	35 (28.23%)
	Unknown	27 (21.77%)
Family history		
	Yes	30 (24.19%)
	No	94 (75.81%)

**Figure 1:**
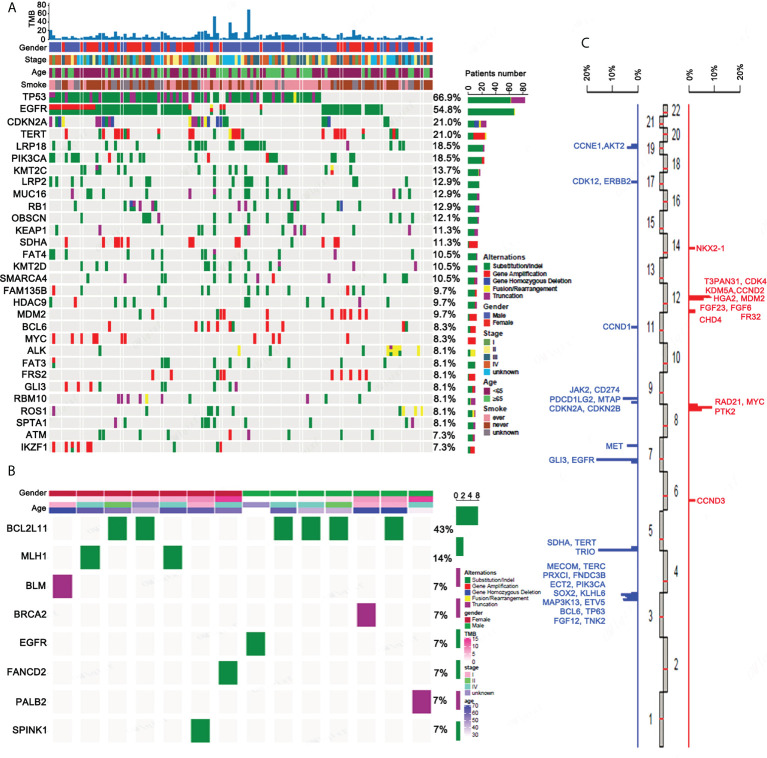
The genetic mutation profiles of ASC patients. **(A)** The top 30 somatic mutational genes containing different forms of mutations as well as their frequencies. **(B)** All germline mutational genes containing different forms of mutation as well as their frequencies. **(C)** The distribution of CNVs on chromosomes.

We performed a panel-based NGS, consisting of 450 cancer-associated genes (**Supplementary Table 1**) in tissue samples obtained from 124 ASC patients, in order to assess molecular profiles. Mutations, including short nucleotide variations (SNVs), long nucleotide variations (LONGs), copy number variations (CNVs), and gene-gene fusions (FUSs), were determined. For the total 1,962 mutations elucidated, 1,415 were SNV, 48 were LONG, 427 were CNV, and 64 were FUS. The top 30 mutated genes determined in ASC are provided in **Figure 1A**. *TP53* and *EGFR* were the most prevalent somatic mutations observed, at 66.9% and 54.8%, respectively, followed by *CDKN2A* (21%), *TERT* (21%), and *LRP1B* (18.5%). Mutations for *BCL2L11* (43%), *MLH1* (14%), *BLM* (7%), *BRCA2* (7%), *EGFR* (7%), FANCD2 (7%), PALB2 (7%), and SPINK1 (7%) are all germline mutations (**Figure 1B**). The distributions for CNVs on chromosomes are provided in **Figure 1C**. The highest incidence for CNVs was found for chromosomes 3 and 12.

### Gene fusion, *EGFR* mutation, and the mutated pathway analysis of ASC

**Figure 2:**
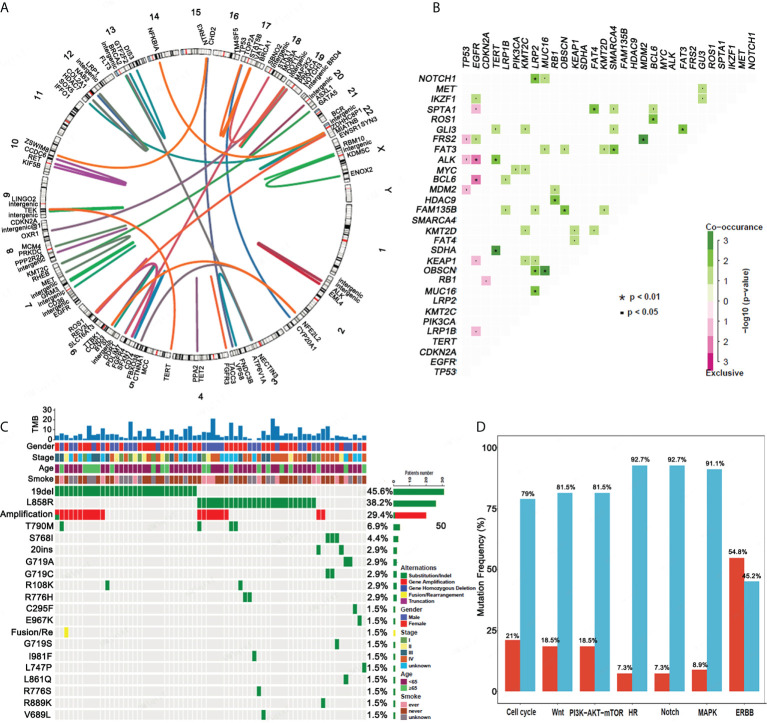
Gene fusion, co-mutation, *EGFR* mutations and mutational gene pathways for ASC. **(A)** An analysis of the gene fusion/rearrangement characteristics for ASC patients. **(B)** The co-occurrence of genomic alterations in ASC patients. **(C)** The frequency of *EGFR* mutations amongst ASC patients. **(D)** The frequency of mutational genes in pathways.

An analysis of the gene fusion/rearrangement characteristics for 124 ASC revealed a total of 64 gene fusions, 15 of which were transchromosomal fusions. The *ALK* fusion had the highest frequency (8/64) of the variants. Six *ALK-EML4* classical fusions were determined as well as two intergenic *ALK* fusions. We also found a *ROS* fusion (4/64) in the form of three *CD74-ROS1* fusions and one *ROS1-SYN3* fusion (**Figure 2A**).

In patients with ASC, *EGFR* mutations were mutually exclusive for both the *ALK* and *BCL6* mutations. *TERT* mutations significantly co-occurred with *ALK* and *SDHA* mutations. *NOTCH1*, *OBSCN*, and *MUC16* mutations co-occurred with *LRP2* mutations, and *NKX2-1* mutations co-occurred with *ERBB2*, *FRS2*, and *RB1* mutations. *FAM135B* and *MUC16* mutations were determined to co-occur with the *OBSCN* mutation, and the *RB1* mutation significantly co-occurred with the *HDAC9* mutation. *FAT4* mutations significantly co-occurred with *SPTA1* mutations. *FAT3* mutations co-occurred with the *SMARCA4* and *GLI3* mutations, and *FRS2* mutations co-occurred with *MDM2* mutations. *ROS1* mutations co-occurred with *BCL6* mutations (**Figure 2B**).

*EGFR* mutations were detected in 68 patients. The top five *EGFR* mutations in mutation frequency in turn are *EGFR* 19del (45.6%), *EGFR* L858R (38.2%) and its amplification (29.4%), T790M (5.9%), and S768I (4.4%) (**Figure 2C**). Of 68 patients, 35 (51.47%) had a single *EGFR* mutation, including 19 (27.94%) with *EGFR* 19del. Eleven (11,16.18%) patients had an *EGFR* L858R mutation in exon 21. A single rare mutation, G719A, C295F, E967K, L747P, and 20 ins were each found in one patient. Complex mutations consisting of 19 del or L858R + T790M were found in four patients. Complex mutations consisting of 19 del or L858R plus other rare mutations were determined in 24 patients. Other rarely reported uncommon mutations included G719A + L861Q (n = 1), G719C + S768I (n = 2), G719S + S768I (n = 1), and R889K + amplification (n = 1). *EGFR* mutation types determined from our analysis are presented in **Table 2**.

**Table 2:** EGFR mutation type in our analysis.

EGFR mutation	Classification	Results, n (%)
Single mutation		35 (51.47%)
	19 del	19 (27.94%)
	L858R	11 (16.18%)
	20 ins	1 (1.47%)
	C295F	1 (1.47%)
	E967K	1 (1.47%)
	G719A	1 (1.47%)
	L747P	1 (1.47%)
Complex mutation		33 (48.53%)
	19 del + amplification	8 (11.70%)
	19 del + amplification + fusion	2 (2.90%)
	19 del + R108K	1 (1.47%)
	19 del + T790M + amplification	1 (1.47%)
	20 ins + amplification	1 (1.47%)
	G719A + L861Q	1 (1.47%)
	G719C+ S768I	2 (2.94%)
	G719S+ S768I	1 (1.47%)
	L858R + amplification	6 (8.82%)
	L858R + R108K	1 (1.47%)
	L858R + R776S	1 (1.47%)
	L858R + T790M	2 (2.94%)
	L858R + T790M + amplification	1 (1.47%)
	L858R + V689L	1 (1.47%)
	L858R + R776H	2 (2.94%)
	R889K + amplification	1 (1.47%)
	L858R + I981F	1 (1.47%)

We also analyzed the pathway of mutated genes in 124 patients with ASC and determined that a minimal percentage of mutated cases was observed for the HR and Notch pathways of ASC (7.3%), whereas a maximum percentage was observed for the ERBB signaling pathway (54.8%) (**Figure 2D**).

### The mutational signature distribution in ASC

**Figure 3:**
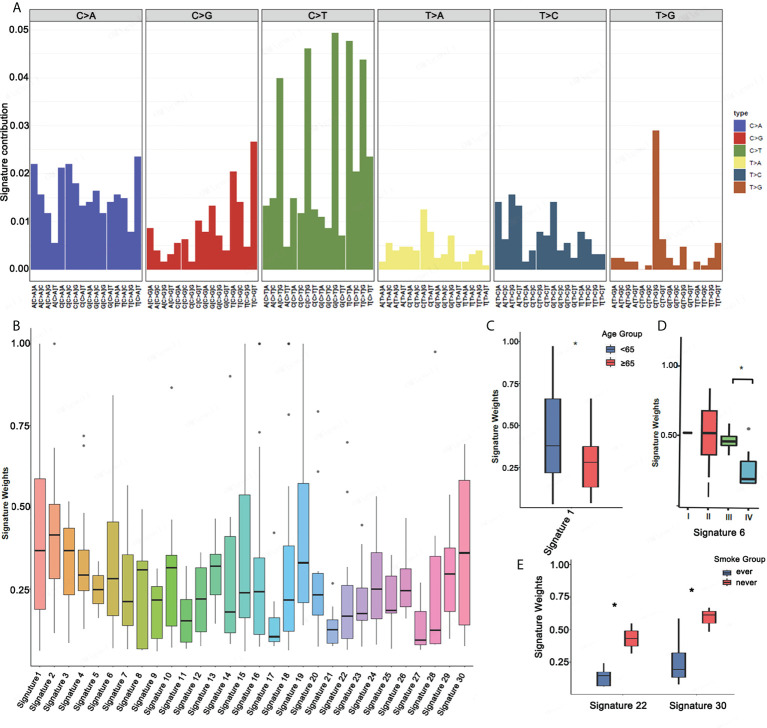
Mutational signatures in ASC patients. **(A)** Mutational activities for the corresponding extracted mutational signatures. **(B)** Mutational activities for the corresponding displayed mutational signatures. **(C–E)** The association between mutational signature and age, stage, and smoking *p <0.05.

**Figure 4:**
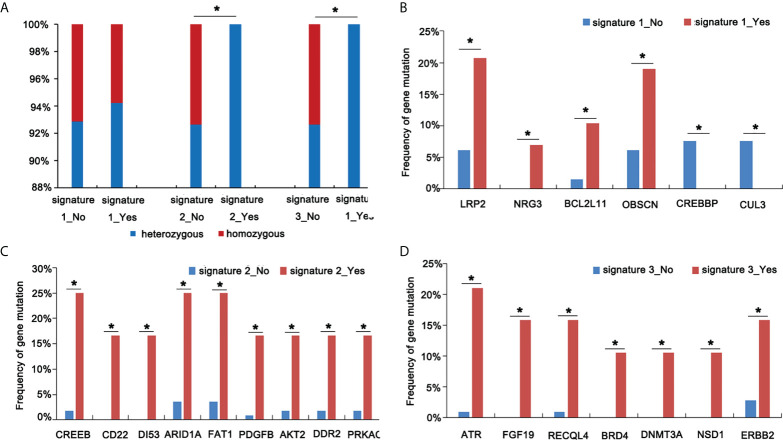
Screen mutational genes in patients having Signatures 1, 2, and 3. **(A)** The analysis of HLA status in patients with or without Signatures 1, 2, and 3. **(B)** The analysis of gene mutations in patients with or without Signature 1. **(C)** The analysis of gene mutations in patients with or without Signature 2. **(D)** The analysis of gene mutations in patients with or without Signature 3 *p < 0.05.

Mutational signatures may reflect specific underlying factors related to tumor development. Therefore, we delineated mutation signatures based on somatic mutation data to evaluate whether these subgroups were characterized by different mutational spectra. To determine mutational signatures in ASC patients, we analyzed NGS data and extracted mutational signatures in accordance with the COSMIC database that included 30 defined signatures. For ASC patients, we determined that most high-frequency base changes were C > T (**Figure 3A**). The five most frequent signatures for patients, ordered in frequency from highest to lowest, were 2, 3, 1, 30, and 13 (**Figure 3B**). Signature 1 was found to be associated with age and Signature 6 was assumed to be associated with a defective stage in ASC (**Figure 3C** and **Figure 3D**). We also observed that Signatures 22 and 30 were significantly related to smoking (**Figure 3E**). We additionally determined that all 13 patients with Signatures 2 and 3 were HLA-B heterozygous, while some patients without Signatures 2 and 3 were HLA-B homozygous (**Figure 4A**). We also analyzed differences in gene mutations between patients with Signature 1, 2, or 3 and without Signature 1, 2, or 3 and found the frequency of several mutations were significantly higher in patients with Signature 1, 2, or 3 than that in patients without Signature 1, 2, or 3 (**Figures 4B–D**).

### The assessment of TMB and PD-L1 in ASC

**Figure 5:**
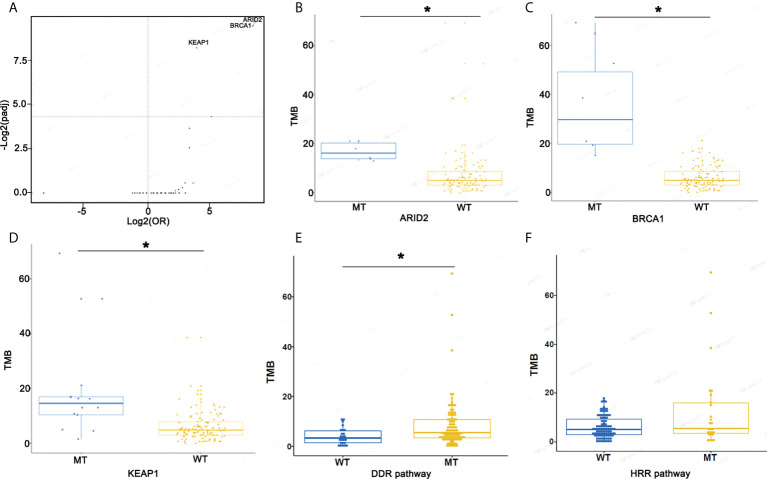
Screen mutational genes and pathways related to TMB. **(A)** The association of mutational genes and TMB. **(B)** TMB in patients with *ARID2*-MT and *ARID2*-WT. **(C)** TMB in patients with *BRCA1*-MT and *BRCA1*-WT. **(D)** TMB in patients with *KEAP1*-MT and *KEAP1*-WT. **(E, F)** TMB in patients with WT or MT in the DDR and HRR pathways *p < 0.05.

To evaluate genomic variations and their correlation to TMB, we analyzed patients with lung ASC and determined that mutations *ARID2*, *BRCA1*, and *KEAP1* were associated with high TMB (**Figures 5A–D**). The TMB index was higher in patients with mutant-type *ARID2*, *BRCA1*, or *KEAP1* as compared to patients with wild-type *ARID2*, *BRCA1*, or *KEAP1*. The ASC patients with homologous recombination repair (HRR) pathway-related gene mutations had a slightly higher TMB as compared to patients without HRR gene mutations; patients with DNA damage response (DDR) pathway mutations had a significantly higher TMB as compared to patients without DDR pathway mutations (**Figures 5E, F**).

We also assessed the expression of PD-L1 in ASC patients. We performed a univariate analysis for the association between PD-L1 expression (evaluated as categorical variables with cut-off values of 1% and 50%) and clinical features of NSCLC. A total of 68 ASC patients were tested for PD-L1 expression. Of these patients, 50.8% were PD-L1 positive, 7.4% had a TPS ≥ 50%, and 47% had a TPS between 1 and 49% (**Supplementary Figure 1**). Our data is generally consistent with reported PD-L1 positivity in ADC.

## Discussion

ASC is a relatively rare tumor with strong aggressiveness and poor prognosis. Surgery is the first choice and the main treatment for ASC. Surgical procedures for ASC are similar to other types of NSCLC. Although the postoperative survival rate of ASC patients has improved, the prognosis for ASC is still not satisfactory as compared to ADC and SCC (5). The postoperative, cumulative, 5-year survival rate of patients with ASC is significantly lower as compared to the survival rate of patients with other types of carcinomas (6.2% *vs*. 41.5%, respectively) (3). Continued studies are required in order to investigate genetic alterations and to explore potential therapies for lung ASC. Our study is the first report to provide comprehensive analyses for genomic features and mutational signatures in lung ASC.

Previous studies have indicated that the most frequent alterations in 28 surgically resected ASCs include *EGFR* (79%), *TP53* (68%), and *MAP3K1* (14%) mutations, and *EGFR* (32%) and *MDM2* amplifications (18%) (7). Our study identified *TP53* and *EGFR* as the most prevalent somatic mutations, present in 66.9% and 54.8% of cases, respectively, followed by *CDKN2A* (21%), *TERT* (21%) and *LRP1B* (18.5%). In the past, the *EGFR* mutation rate for ASC has been reported to range from 13% to 48% (6, 13–17). Our study yielded a higher frequency for *EGFR* mutations in lung ASC of 54.8%, a value similar to result reported by Cheng et al. (17) who reported a value of 48%. However, in contrast to our findings, a lower prevalence of *EGFR* mutations, 13%, has been reported for lung ASC in the Caucasian ethnic group (18). Several case studies have indicated good therapeutic responses in patients taking *EGFR* inhibitors (19–21). In our study, *EGFR* mutations occur in 83.72% (36/43) of non-smokers and *EGFR* mutations occur in 25% of smokers, suggesting that the high proportion of patients with harboring EGFR mutations is due to the high proportion of non-smokers. *EGFR*-TKIs should be considered as first-line therapies for patients with *EGFR* mutant ASC.

Several experiments supported a pivotal role for *EML4–ALK* in lung cancer. For example, a specific inhibitor for *ALK* induced rapid cell death in one NSCLC cell line, NCI-H3122, harbors variant 1 of *EML4–ALK* (22). *EML4-ALK* appears to be a new oncogene involved in NSCLC, particularly in nonsmokers (23). We found 64 gene fusions in ASC patients. The highest frequency for variants was determined to occur for *ALK* fusions in six *ALK-EML4* classical and two intergenic *ALK* fusions. We also identified five *ALK-EML4* classical and two intergenic *ALK* fusions in nonsmokers. Our data indicate that *ALK* fusions may be associated with nonsmokers. Based on our results, we conclude that *EML4-ALK* may be useful for predicting potential responses to *ALK* inhibitors used as therapeutic options for patients with lung cancer.

Somatic mutations in cancer genomes are caused by multiple mutational processes, each of which generates a characteristic mutational signature (24). The analysis of mutational signatures is becoming routine in cancer genomics and has implications for pathogenesis, classification, and prognosis. Amongst signatures cataloged by COSMIC, the most high-frequency base changes we observed were C > T. The five most frequent signatures in ASC, from highest to lowest, were 2, 3, 1, 30, and 13. Signature 4 was enriched in ADC and SCC. Most of these signatures are attributed to smoking (10, 25). In ADC, Signatures 2 and 13 display a higher number of mutations in smokers as compared to non-smokers (26). We observed that Signatures 22 and 30 were significantly related to smoking. The mutation signatures found in almost all cancer types were Signature 6 related to mismatch repair deficiency and Signature 1 that reflects the natural decomposition of 5-methylcytosine into thymine associated with aging (9). In line with previous studies, our study revealed that Signatures 1 and 6 are associated with age and tumor stage, respectively. The analysis of mutational signatures, to some extent, revealed a correlation between ASC and clinical features. Specificity may be applied in order to determine the unique underlying mechanism of occurrence and development for ASC.

There are some limitations in this study as follows: 1) the data of alterations including DNA and RNA subclonality between each component (ADC and SCC) within the same tumor need further explored in the future; 2) In our study, the ADC component of tumors varied between 10% and 90% (mean, 60.3%), and the SCC component of tumors varied between 10% and 80% (mean, 30.6%). In the reported series of ASC, there were 68 ADC-predominant cases, 42 SCC-predominant cases, and 14 cases with equal ADC and SCC. The different profiles depending on the proportion of ADC and SCC components, including PD-L1, such as more gene mutations in tumors with a high ADC component were remain unclear, which also will be developed in the future studies.

We assembled and characterized genomic data and mutational signatures from 124 ASC patients in order to determine whether or not tumor genetic landscape impacts clinical benefits. We identified genomic mutation signatures and molecular biomarkers in ASC that may provide theoretical insights for clinicians making treatment plans for lung ASC patients.

## Data availability statement

The raw data supporting the conclusions of this article will be made available by the authors, without undue reservation.

## Ethics statement

The studies involving human participants were reviewed and approved by the Committee for Ethical Review of Research at Cancer Hospital of Shantou University Medical College (2021090). The patients/participants provided their written informed consent to participate in this study.

## Author contributions

Project development: HW, JL, YC, and YL. Data collection: SZ, KM, and ZL. Data analysis: XQ, LH, LG, and YW. Manuscript writing/editing: HW, JL, YC, YL, SZ, KM, and ZL. All authors contributed to the article and approved the submitted version.

## Conflict of interest

Author XFQ, LJH, LJG, and YW are employed by OrigiMed Co., Ltd.

The remaining authors declare that the research was conducted in the absence of any commercial or financial relationships that could be construed as a potential conflict of interest.

## Publisher’s note

All claims expressed in this article are solely those of the authors and do not necessarily represent those of their affiliated organizations, or those of the publisher, the editors and the reviewers. Any product that may be evaluated in this article, or claim that may be made by its manufacturer, is not guaranteed or endorsed by the publisher.

## Supplementary material

Supplementary Figure 1 PD-L1 expression in ASC patients.
