# Supplementary figures and images for: Comprehensive analyses of genomic features and mutational signatures in adenosquamous carcinoma of the lung
Source: Front Oncol. 2022 Sep 14;12:945843. doi: 10.3389/fonc.2022.945843 (PMC9518956; doi:10.3389/fonc.2022.945843)

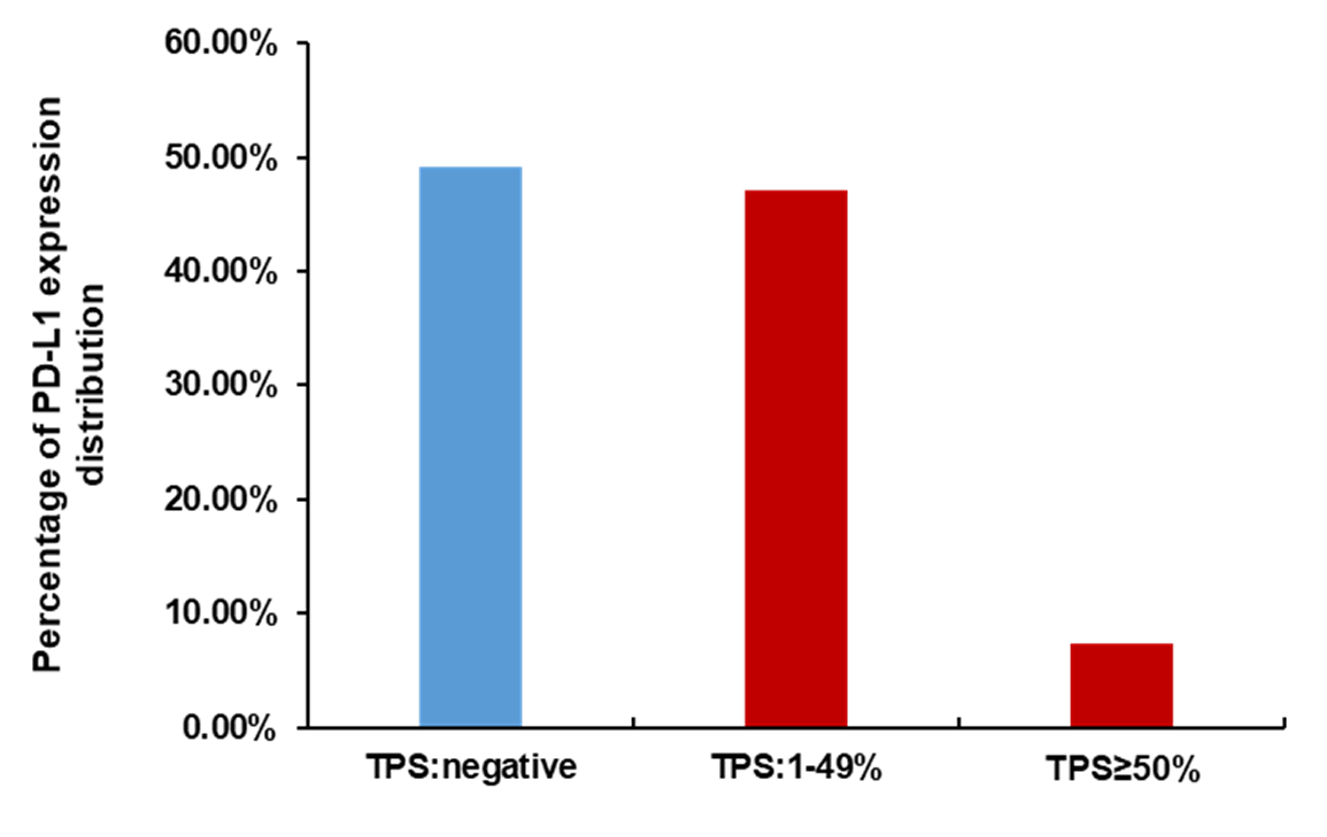

Supplement: Supplementary Figure 1 — PD-L1 expression in ASC patients. [file Image_1.tif]
