# Supplementary material for: Comprehensive analyses of genomic features and mutational signatures in adenosquamous carcinoma of the lung
Source: Front Oncol. 2022 Sep 14;12:945843. doi: 10.3389/fonc.2022.945843 (PMC9518956; doi:10.3389/fonc.2022.945843)
Supplement: Supplementary file 2 [file Table_1.docx]

Supplementary Table 1 Gene lists for OM 450-gene panel.

| **Gene lists for 450-gene** | | | | | | | | |
| --- | --- | --- | --- | --- | --- | --- | --- | --- |
| ABL1 | BTK | DDR1 | FGF23 | IDH1 | MED12 | PARP3 | RANBP2 | STAT4 |
| ABL2 | CAMTA1 | DDR2 | FGF3 | IDH2 | MEF2B | PARP4 | RARA | STK11 |
| ACVR1B | CARD11 | DICER1 | FGF4 | IGF1R | MEN1 | PAX5 | RB1 | STK24 |
| ACVR2A | CBFB | DNMT3A | FGF6 | IGF2 | MERTK | PBRM1 | RBM10 | SUFU |
| ADAM29 | CBL | DOT1L | FGF7 | IKBKE | MET | PCA3 | RECQL | SYK |
| ADGRA2 | CCND1 | DPYD | FGFR1 | IKZF1 | MGMT | PDCD1 | REL | TAF1 |
| AKT1 | CCND2 | EGF | FGFR2 | IL7R | MITF | PDCD1LG2 | RELA | TBX3 |
| AKT2 | CCND3 | EGFR | FGFR3 | INHBA | MLH1 | PDGFB | RELB | TCF7L2 |
| AKT3 | CCNE1 | EMSY | FGFR4 | INPP4B | MPL | PDGFRA | RET | TEK |
| ALK | CD274 | EP300 | FGR | IRF2 | MRE11 | PDGFRB | RHBDF2 | TERT |
| AMER1 | CD79A | EPCAM | FH | IRF4 | MS4A1 | PDK1 | RHOA | TET1 |
| APC | CD79B | EPHA2 | FLCN | IRS2 | MSH2 | PIK3C2B | RICTOR | TET2 |
| APEX1 | CDC73 | EPHA3 | FLI1 | ITK | MSH6 | PIK3CA | RNF43 | TET3 |
| AR | CDH1 | EPHA5 | FLT1 | JAK1 | MST1R | PIK3CB | ROCK1 | TFE3 |
| ARAF | CDK12 | EPHA7 | FLT3 | JAK2 | MTOR | PIK3CD | ROCK2 | TGFBR1 |
| ARFRP1 | CDK4 | EPHB1 | FLT4 | JAK3 | MUTYH | PIK3CG | ROS1 | TGFBR2 |
| ARID1A | CDK6 | ERBB2 | FOS | JUN | MYB | PIK3R1 | RPTOR | TIE1 |
| ARID1B | CDK8 | ERBB3 | FOXL2 | KAT6A | MYC | PIK3R2 | RUNX1 | TIPARP |
| ARID2 | CDKN1A | ERBB4 | FOXO1 | KDM5A | MYCL | PKD2 | RUNX1T1 | TMPRSS2 |
| ASXL1 | CDKN1B | ERCC1 | FOXP1 | KDM5B | MYCN | PLA2G1B | RXRA | TNFAIP3 |
| ATF1 | CDKN2A | ERG | FRS2 | KDM5C | MYD88 | PLCG2 | SDHA | TNFRSF14 |
| ATM | CDKN2B | ERRFI1 | FUBP1 | KDM6A | NBN | PMS2 | SDHB | TNFSF11 |
| ATR | CDKN2C | ESR1 | FUS | KDR | NCOA2 | POLB | SDHC | TNFSF13B |
| ATRX | CEBPA | ETV1 | FYN | KEAP1 | NCOR1 | POLD1 | SDHD | TNK2 |
| AURKA | CFTR | ETV4 | GABRA6 | KEL | NEK11 | POLE | SETD2 | TOP1 |
| AURKB | CHD2 | ETV5 | GATA1 | KIT | NF1 | PPP2R1A | SF3B1 | TOP2A |
| AXIN1 | CHD4 | ETV6 | GATA2 | KLHL6 | NF2 | PRDM1 | SIK1 | TP53 |
| AXIN2 | CHEK1 | EWSR1 | GATA3 | KMT2A | NFE2L2 | PREX2 | SLIT2 | TPMT |
| AXL | CHEK2 | EZH2 | GATA4 | KMT2C | NFIB | PRKACA | SMAD2 | TSC1 |
| BAP1 | CIC | FAM135B | GATA6 | KMT2D | NFKBIA | PRKAR1A | SMAD3 | TSC2 |
| BARD1 | COL1A1 | FAM46C | GID4 | KRAS | NKX2-1 | PRKCI | SMAD4 | TSHR |
| BCL2 | CRBN | FANCA | GLI1 | LCK | NOTCH1 | PRKDC | SMARCA4 | TYK2 |
| BCL2L1 | CREB3L1 | FANCC | GLI2 | LIMK1 | NOTCH2 | PRSS1 | SMARCB1 | U2AF1 |
| BCL2L11 | CREB3L2 | FANCD2 | GLI3 | LMO1 | NOTCH3 | PRSS8 | SMARCD1 | UGT1A1 |
| BCL2L2 | CREBBP | FANCE | GNA11 | LRP1 | NOTCH4 | PTCH1 | SMO | VEGFA |
| BCL6 | CRKL | FANCF | GNA13 | LRP1B | NPM1 | PTEN | SNCAIP | VHL |
| BCOR | CRLF2 | FANCG | GNAQ | LRP2 | NR4A3 | PTK2 | SOCS1 | WEE1 |
| BCORL1 | CSF1 | FANCL | GNAS | LYN | NRAS | PTK6 | SOX10 | WEE2 |
| BCR | CSF1R | FANCM | GRIN2A | LZTR1 | NRG1 | PTPN11 | SOX2 | NSD2 |
| BIRC5 | CSK | FAS | GRM3 | MACC1 | NRG3 | QKI | SOX9 | WISP3 |
| BLK | CSNK1A1 | FAT1 | GSK3B | MAGI2 | NSD1 | RAC1 | SPEN | WT1 |
| BLM | CTCF | FAT3 | H3F3A | MAP2K1 | NTRK1 | RAD50 | SPINK1 | XIAP |
| BMPR1A | CTNNA1 | FAT4 | HCK | MAP2K2 | NTRK2 | RAD51 | SPOP | XPO1 |
| BMX | CTNNB1 | FBXW7 | HDAC9 | MAP2K4 | NTRK3 | RAD51B | SPTA1 | XRCC2 |
| BRAF | CUL3 | FEN1 | HGF | MAP3K1 | NUP93 | RAD51C | SRC | XRCC3 |
| BRCA1 | CXCR4 | FEV | HNF1A | MAP3K13 | PAK3 | RAD51D | SRMS | YES1 |
| BRCA2 | CYLD | FGF10 | HRAS | MAP4K5 | PALB2 | RAD52 | SS18 | ZBTB2 |
| BRD4 | CYP17A1 | FGF12 | HSD3B1 | MCL1 | PARK2 | RAD54B | SSX1 | ZNF217 |
| BRIP1 | CYP2D6 | FGF14 | HSP90AA1 | MDM2 | PARP1 | RAD54L | STAG2 | ZNF703 |
| BTG1 | DAXX | FGF19 | HTATIP2 | MDM4 | PARP2 | RAF1 | STAT3 | ZNF750 |
